# Intensive Speech Therapy for Hypokinetic Dysarthria in Parkinson’s Disease: Targeting the Five Subsystems of Speech Production with Clinical and Instrumental Evaluation

**DOI:** 10.3390/neurosci6010007

**Published:** 2025-01-16

**Authors:** Annalisa Gison, Marco Ruggiero, Davide Tufarelli, Stefania Proietti, Daniela Moscariello, Marianna Valente

**Affiliations:** 1 IRCCS San Raffaele Roma, 00163 Rome, Italy; 2 Physical and Rehabilitation Medicine 2, IRCCS Istituto Ortopedico Rizzoli, 40136 Bologna, Italy

**Keywords:** speech therapy, hypokinetic dysarthria, Parkinson disease, voice analysis, IOPI

## Abstract

*Background*: Hypokinetic dysarthria is a speech disorder observed in almost 90% of PD patients that can appear at any stage of the disease, usually worsening as the disease progresses. Today, speech therapy intervention in PD is seen as a possible therapeutic option to alleviate and slow down the progression of symptoms. This study aims to investigate the validity of traditional speech therapy in dysarthria with the aim of improving the quality of life of PD patients, by comparing subjective clinical assessment with objective instrumental measures (IOPI and voice analysis). *Methods*: This is an observational study of 30 patients with hypokinetic dysarthria due to PD. The patients underwent speech therapy treatment with a frequency of three times per week for 12 consecutive weeks. Patients were evaluated at the time of enrollment (T0), at the start of treatment (T1), and at the end of the same (T2). Six months after the end of treatment (T3), a follow-up was performed based on disability and phonatory evaluation. *Results*: This study showed significant improvements (<0.001) from the start (T1) to the end of treatment (T2), with increases in the Barthel Index score, Robertson Dysarthria Profile, and IOPI measurements for tongue and lip strength, along with enhanced phonometer scores and tongue endurance. Correlations highlighted that tongue endurance decreased with age, CIRS, and MDS-UPDRS, while showing a positive association with MoCA scores. *Conclusions*: Significant improvements were observed in tongue and lip strength, phonatory duration, intensity, and vocal quality between pre- (T1) and post-treatment (T2). This study underscores the importance of early and continuous speech therapy treatment for comprehensive speech function enhancement.

## 1. Introduction

In the classification of dysarthria, voice and speech disorders in Parkinson’s disease (PD) are defined as “hypokinetic dysarthria” [1].

Hypokinetic dysarthria is a perceptually distinct motor speech disorder (MSD) associated with basal ganglia control circuit pathology. It can occur at any or all levels of respiratory, phonatory, resonatory, and articulatory speech, but its characteristics are most evident in voice, articulation, and prosody [2]. Physiologically, the disorder reflects the effects of stiffness, reduced strength and range of motion, and slow individual but sometimes fast repetitive movements on speech. Reduced range of motion contributes significantly to the disorder, hence its designation as hypokinetic dysarthria [3].

These features, together with amimia, which consists of a reduced facial expression, contribute to limiting the communication of most patients with PD [3] and impairing the intelligibility of speech with significant effects in psychological, social, and occupational settings [4], thus reducing quality of life leading also to social isolation [5].

Although dysarthria usually does not emerge for several years after the first signs of PD, it becomes evident during the disease course, nearly always preceding the onset of dysphagia, which occurs in 40% of cases [6]. Until a few years ago, speech therapy in PD was rarely prescribed and only in advanced stages, when the patient’s intelligibility was already severely compromised, with the aim of reducing the risk of aspiration due to dysphagia [7]. Today, speech therapy in PD is seen as a possible therapeutic option, from the early stages of the disease, within a comprehensive program to alleviate and slow down the progression of symptoms, particularly in dysarthria [4,8].

When planning rehabilitative treatments for dysarthria, it is necessary to identify speech characteristics and their relationship with each of the productive subsystems. Changes can be observed in both the phonatory and articulatory subsystem; therefore, it is important to correlate speech characteristics with dysfunction in various muscle groups to help determine which component of the system deserves attention. Furthermore, treatment of dysarthria requires a good degree of patient cooperation [9].

In this context, acoustic analysis of voice, using an electronic system, allows the identification of changes in voice parameters for predicting the disease worsening and for targeting specific intervention. Among the voice parameters, jitter and shimmer significantly increased in patients with PD [10]. In addition, tongue strength and endurance are significantly reduced in PD, and could be a sensitive indicator of disease progression [11].

Several studies compare voice disorders in patients in PD before and after pharmacological treatment, showing a noticeable reduction in the severity of voice disorders following levodopa, with a subsequent worsening once the drug effect wears off [12,13]. Since drugs have only modest and temporary effects on Parkinsonian speech, behavioral treatments are generally preferred [14].

Although many authors analyzed voice parameters and assessed suprahyoid muscles and tongue strength [15,16,17], few studies have investigated both aspects within the same study. Marchese et al. investigated the effectiveness of LSVT®, intensive voice treatment in improving prosody in patients with PD, analyzing the acoustic parameters related to prosody [18]. A recent meta-analysis suggests a beneficial effect of SLT in improving perceptual intelligibility, sound pressure level, and semitone standard deviation [19].

Bearing this in mind, the aim of the present study was to evaluate the clinical and functional improvement in patients with hypokinetic dysarthria due to Parkinson’s disease following intensive speech therapy, assessed through the measurement of tongue muscle strength and voice analysis [20].

## 2. Materials and Methods

This is an observational pilot study conducted at IRCCS San Raffaele Neuromotor Day Hospital in Rome, where 30 patients affected by PD were enrolled from July 2018 to January 2021.

The study protocol underwent local ethics committee approval (“IRCCS San Raffaele Roma” Ethic Committee protocol code RP SR19/18). Written informed consent was obtained from all the participants. This study was conducted in conformity with the ethical standard according to the Declaration of Helsinki.

### 2.1. Subjects

All patients underwent a standard speech clinical assessment. Voluntary patients with Parkinson’s disease grade I–IV of the Hoehn and Yahr staging [20] were included in this study, with these inclusion criteria:

- Patients with functional communicative intentionality;
- Patients with mild to severe dysarthria, according to the clinical evaluation of PRM physician;
- Montreal Cognitive Assessment (MoCA) [21,22] with threshold score ≥ 15.5.

Conversely, we excluded those patients with:

- Severe hearing loss and/or hearing aid wearers;
- Inability to provide informed consent;
- Comorbidity with other neurological and/or vocal pathologies;
- Temporo-mandibular joint (TMJ) disorder;
- Myofascial pain syndrome;
- Demolitive/reconstructive surgical treatment for neoplasms of oral cavity and oropharynx;
- High risk of seizures.

### 2.2. Study Design

**Figure 1:**
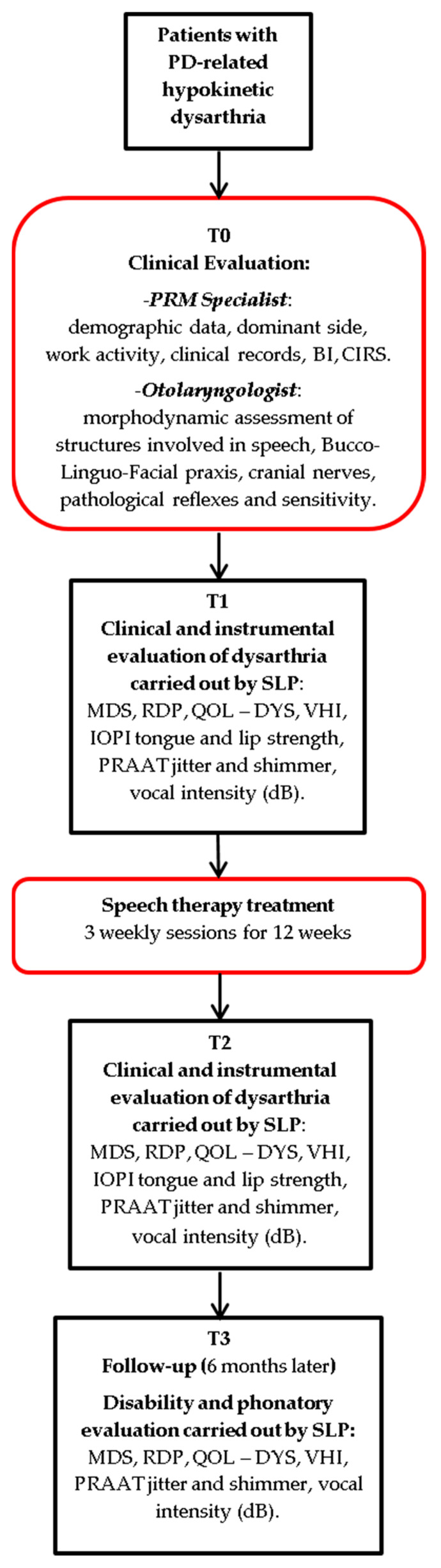
Study flow-chart.

Patients were evaluated at the time of enrollment (T0), at the start of treatment (T1), and at the end of the same (T2). Six months after the end of treatment (T3), a follow-up was performed based on disability and phonatory evaluation (Figure 1).

At T0, all of the patients underwent clinical evaluation with a Physical and Rehabilitation Medicine (PRM) Specialist and an Otolaryngologist, as is usual care for our institution. At T1 and T2, a clinical and instrumental evaluation was performed by a speech and language pathologist. At T3, a follow-up was performed to assess the impact on disability related to dysarthria.

### 2.3. Speech Therapy Treatment

The patients underwent a speech therapy treatment consisting of 36 sessions, with a frequency of 3 times per week and a duration of 40 min, for 12 consecutive weeks.

Since speech articulation is the result of the functioning of five distinct systems (respiratory, phonatory, resonance, articulatory, prosodic), speech therapy focused on different types of exercises specific to each subsystem [6] (Table 1).

### 2.4. Clinical Evaluation

A PRM assessment was necessary to detect disability and the need for rehabilitation treatment [23]. In this evaluation, demographic data (gender, date of birth, education, dominant side, work activity, presence of social network), clinical data (comorbidities, drug therapy, date of disorder onset), and scores of specific assessment scales, Barthel Index to establish the degree of functional independence [24,25,26] and Cumulative Illness Rating Scale (CIRS) to measure physical illness burden [27], of all patients were collected.

The severity and progression of Parkinson’s disease were assessed using the Hoehn and Yahr scale [28]. The Hoehn and Yahr scale is a staging system for Parkinson’s disease based on the severity of motor symptoms, ranging from stage 1 (unilateral symptoms) to stage 5 (total disability). The scale primarily considers motor asymmetry, with the affected side initially exhibiting symptoms unilaterally, which become bilateral as the disease progresses. It is used to monitor disease progression and guide therapeutic management [28,29].

At T0, morphodynamic assessment of the structures involved in speech, evaluation of Bucco-Linguo-Facial praxis, cranial nerve function, assessment of pathological reflexes, and sensitivity was made by an Otolaryngologist.

### 2.5. Speech Therapist Assessment

The phases of the assessment process include the anamnesis, evaluation of the phono-articulatory organs, perceptual evaluation of the phono-articulatory product, and speech intelligibility.

Speech analysis was conducted according to RDP [30,31], to assess the patient’s level of disability in various aspects: breathing, phonation, facial muscles, diadochokinesis, reflexes, articulation, intelligibility, and prosody. Conversational speech or reading, rapid execution of alternating movement patterns, and vowel prolongation provide useful information about salient and distinguishing speech characteristics (monopitch, monoloudness, reduced stress, short phrases, variable rate, short rushes of speech, imprecise consonants, inappropriate pauses, tachyphemia) [1,2].

The functional scales were administered at the start (T1), at the end of treatment (T2), and at the follow-up (T3) six months after the end of the treatment, by a speech and language therapist as follows:

- MDS—Unified Parkinson Disease Rating Scale (MDS-UPDRS) [32], to evaluate various aspects of Parkinson’s disease including non-motor and motor experiences of daily living and motor complications with ratings on a 5-point Likert scale (from 0 Normal to 4 Severe).
- Robertson Dysarthria Profile (RDP) [30], aims to identify the patient’s level of disability in the various aspects: breathing, phonation, facial musculature, diadochokinesis, reflexes, articulation, intelligibility, and prosody with ratings on a 4-point Likert scale (from 1 Poor to 4 Excellent). In this way, highlighting the individual’s difficulties in carrying out some activities, it was possible to outline a treatment framework aimed at recovering residual potential and therefore minimizing the associated disability.
- QOL—DYS: Dysarthria self-assessment questionnaire [33], which consist of 4 sections of 10 items each, with ratings on a 5-point Likert scale (from 0 Never to 4 Always).
- Voice Handicap Index (VHI) [34], to assess dysphonia-related quality of life. This index addresses the impact of vocal issues on daily activities, the psychological impact, and the perception of vocal emission characteristics, with ratings on a 5-point severity scale (0–4).

### 2.6. Instrumental Assessment

Dysarthria may lead to alterations in the strength, speed, amplitude, stability, pitch, and accuracy of the movements in each of the speech subsystems [6]. For this reason, for the instrumental evaluation we selected tools that specifically assess and record these parameters.

The following devices and tools were used:

- IOPI (Iowa Oral Performance Instrument) [35];
- PRAAT software v. 6.4.24, shimmer (percentage) and jitter (percentage) [36];
- Sound level recorder (fonometro SL400) [37].

The instrumental assessment was performed at all timepoints, except for the use of the IOPI, which was applied only at T1 and T2, before and after treatment, and not during the 6-month follow-up (T3).

The IOPI device was used to measure tongue and lip strength and endurance. Patients pushed their tongue as hard as they could against the air-filled IOPI bulb for approximately 2 s to measure tongue strength, and as long as possible to measure tongue endurance. Next, the patients pressed the bulb against their teeth while clenching their lips tightly for about 2 s to measure lip strength, and as long as possible to measure lip endurance. Then, the patients were allowed to rest for 30–60 s, and then repeated each test twice.

To record the psycho-acoustic parameters of the voice, evaluate the patient’s intelligibility, and complete the non-verbal evaluation, a sound level recorder and the PRAAT were used, respectively. With the sound level recorder, we assessed vocal intensity, expressed in decibel (dB). Then, the multi-parametric acoustic analysis was carried out using the PRAAT computerized system.

The software was used to analyze the middle 3 s of a prolongation vowel/a/held at relatively constant pitch and intensity. In a percentage graph, out of 22 acoustic parameters, we considered the following 2 to be the most important in the literature as indicators of vocal alterations in PD [10,36,38]:

(1) Jitt—percentage jitter: it is the short-term, period-to-period average relative variability of the fundamental period. It is an index of the irregularity of the glottic vibration and is perceptually correlated to the hoarse voice;

(2) Shim—percentage shimmer: it is the short-term average relative variability, period by period, of the peak-to-peak amplitude. It is an index of the irregularity of the glottic vibration and is perceptually correlated to the breathy and hoarse voice.

**Figure 2:**
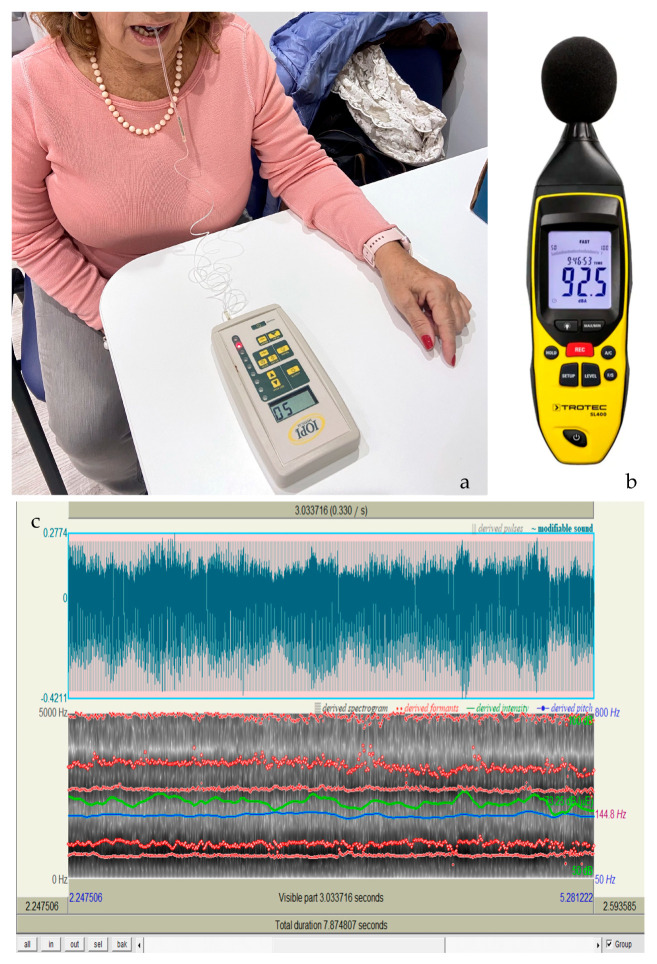
(**a**) Illustrates the use of the IOPI device. (**b**) Shows the sound level meter. (**c**) Displays the spectrogram obtained through voice analysis made by the PRAAT system.

The spectrogram (Figure 2c) is a useful tool for analyzing the acoustic characteristics of hypokinetic dysarthria in Parkinson’s disease, allowing the evaluation of parameters such as fundamental frequency (F0), intensity, formants, and speech rate. Alterations in these parameters, such as reduced pitch variability, low vocal intensity, and articulation difficulties, indicate disease progression. Spectrographic analysis also assesses voice tremor, speech coordination, and speech clarity. These outcomes provide crucial data for early diagnosis, monitoring therapeutic efficacy, and planning personalized interventions in speech therapy [6].

### 2.7. Statistical Analysis

Data collection was carried out with dedicated and anonymized case report forms, and a master log was kept by the principal investigator. Data were stored on a dedicated and secured server, and data analysis was performed using R statistical software for Windows version 3.3.2 “https://cran.r-project.org” accessed on 15 October 2024.

We evaluated all of the data collected to find out whether they followed a normal probability distribution. The graphical evaluation method with the normal probability plot and the Shapiro–Wilks test were used to choose the most appropriate statistical test (parametric or nonparametric). Descriptive statistics of all variables were also evaluated. Central tendency was calculated as either standard deviation (SD) or median with percentile ranks, depending on the nature of the variables.

For the pre–post treatment comparison, if the distribution was normal, paired t-tests were applied; otherwise, the Wilcoxon signed-rank test was used. For comparisons between two groups, if the data followed a normal distribution, unpaired t-tests were conducted; otherwise, the Mann–Whitney U-test was employed. In addition, the analysis employed a linear mixed-effects model to evaluate three key factors: treatment, time, and subject. ANOVA (analysis of variance) was used to assess the significance of the fixed effects in explaining variability in the outcome. Furthermore, a multivariate component (MANOVA) was applied to evaluate these effects across multiple dependent variables. By analyzing these relationships within a single framework, MANOVA offers a more comprehensive understanding of the effects and interactions among the variables. This study was exploratory in nature, aiming to explore a wide range of potential associations and hypotheses, rather than testing specific pre-defined hypotheses. Bonferroni correction was applied to assess significant differences between the time-points. All statistical analyses were performed using SPSS 28.0 software.

## 3. Results

There were 30 Parkinson’s disease patients enrolled with a mean age of 72.5 ± 8.1 years, of which 76.7% were male and 93.3% retired. The disease duration averaged 6.87 years, the CIRS averaged 1.5 ± 0.2, and the Hoehn and Yahr staging indicated predominantly right-sided unilateral involvement in 63.3% of cases, while grade I–IV staging was as follows: stage I 46.7%, stage II 36.6%, stage III 6.7%, and stage IV 10%. The mean of the MDS-UPDRS was 34.5 ± 14.5, the mean of the MoCA was 23.0 ± 3.6, and the mean of the Barthel Index was 71.4 ± 3.6.

Table 2 shows the improvement achieved by patients at the end of treatment (T2) in the different outcome measures analyzed. Table 2 indicates the values at time T1 and T2 of Barthel Index, RDP, tongue strength, lip strength (right and left), vocal intensity, and tongue endurance all had a statistically significant change (*p* < 0.001).

The data depicted in Table 3 show that the values from enrollment T1 to T2 and to T3 of the VHI, of the QOL–DYS, the results of the analysis multiparameter acoustics PRAAT JITT and PRAAT SHIM, and the Maximum Phonation Time (MPT) had a statistically significant improvement at all of the time points.

Table 4 shows the correlations between the measurements with the IOPI tool: the resistance of the tongue is negatively correlated with age, with CIRS, and with the MDS-UPDRS. Lip right strength is positively correlated with MoCA.

## 4. Discussion

The main findings of this study are the clinical and instrumental changes observed between pre- and post-treatment in enrolled patients undergoing traditional speech therapy.

In fact, as reported in Table 2, the statistically significant improvement, measured with the RDP as a whole and the self-assessment scales at T1 and T2, is supported by the results obtained at T1 and T2 through the instrumental evaluation. Among the recruited patients, there is a statistically significant increase in tongue strength and endurance, both left and right lip strength, phonatory duration and intensity, and improvement in the vocal quality. Analyzing RDP, regarding the goal of objectifying the improvements obtained from the pneumo-phono-articulatory point of view, promising results were found. The correlation between IOPI tongue endurance scores and RDP scores for phono-articulation was significant, as were pre- and post-treatment measurements of phonation duration and vocal intensity level (the latter obtained using a phonometer). In the report by Marchese et al. [18], similar improvements in vocal quality after LSVT® treatment were observed, but their study did not investigate the instrumental muscle strength assessment in detail. Similarly, Pu et al. [39], in their meta-analysis, confirmed that LSVT® treatment promotes improvements in vocal quality in Parkinson’s disease patients, but did not examine specific correlations between tongue strength and phonation, as we did. Likewise, Munoz Vigueraz [19], while highlighting the efficacy of speech therapy in hypokinetic dysarthria, did not explore the instrumental tools used in our study to quantify muscle strength and duration in relation to vocal improvements.

Regarding prosody and speech intelligibility, no significant results were obtained by correlating the IOPI measurements with the specific sections of the RDP that analyze these two aspects from a subjective point of view, always to be kept in mind when dealing with dysarthric patients. A plausible explanation for the lack of correlation between the speech and non-speech measures is that reduced tongue strength may need to reach a critical level before detrimental effects on speech are perceived [40]. This is consistent with Pu et al. [39], who suggest that significant improvements are seen in simple parameters like vocal intensity, but that for more complex alterations such as intelligibility, results might not be immediately visible.

Statistically significant results (Table 3) were obtained from the voice and dysarthria self-assessment scales, comparing their administration at T1, T2, and T3, which suggests that the patients, even after 6 months, perceive a maintenance of the vocal benefit obtained at T2, and consequently maintain their communicative intentionality with a positive impact on their quality of life. The observed improvements in vocal quality agree with the findings of Marchese et al. [18], who report lasting benefits following LSVT^®^ treatment, and with the results of Munoz Vigueraz et al. [19], who highlight long-term improvements in communication, although without the specific instrumental assessment used in our study.

Tongue resistance seemed to correlate positively with patient age, comorbidities, and autonomy assessed by UPDRS (Table 4). This may, therefore, indicate the need for early speech-language therapy of these patients, thus avoiding having to wait for the disease to progress to more advanced stages.

However, comparing the other assessment scales with results obtained with the IOPI measurements (T1 and T2), no statistically significant differences were obtained. This leads us to infer that the patient has only partial awareness of the improvements achieved through speech therapy rehabilitation, and that further studies, maybe using biofeedback devices, are needed to enhance this recovery [41].

In conclusion, this is a pilot study aimed at testing the appropriateness and feasibility of the study design to obtain preliminary data for future studies. The results obtained reasonably support the notion that rehabilitation protocols in PD must necessarily include speech therapy treatment from the earliest stages of the disease to maintain adequate oral and interpersonal communication skills [7,8]. Having obtained a non-significant correlation between the IOPI and RDP assessments and the IOPI and PRAAT assessments, we were unable to demonstrate that as the strength and resistance of the tongue and lips increases, the overall speech motor control improves.

The main weakness of the present study was its observational design, as well as the lack of randomization. An additional limitation of this study is the small sample size, combined with the absence of a long-term follow-up after treatment. Furthermore, the patients were recruited with the same inclusion criteria, in the same rehabilitation unit, and then treated by the same rehabilitation group. Thus, future crossover randomized controlled trials are needed to draw more consistent conclusions.

## 5. Conclusions

This study provided evidence that speech therapy can improve, in the short term, the strength of the tongue in patients with hypokinetic dysarthria caused by Parkinson’s disease. It also improves vocal parameters associated with voice quality, such as jitter and shimmer, which are particularly related to hoarseness and breathiness, respectively.

To further demonstrate, through objective data, that speech rehabilitation must be continuous and intensive to achieve functional benefits at the pneumophonic and articulatory levels, additional studies are needed. These studies should involve isometric lingual strength training with the IOPI, combined with traditional isotonic exercises, to assess their effects on vocal function, pneumophonic–articulatory coordination, and swallowing.

## Figures and Tables

**Table 1:** Specific exercises for each speech subsystem. * Intensity increased every 3 weeks.

System	Exercise Description	Duration	Dose/Intensity
Respiratory	•Establish optimal conditions for breathing through relaxation and body posture	1′	3 repetitions
	•Decrease the inhalation time and lengthen the exhalation time with non-verbal exercises	2′	5 repetitions
Phonatory (for pneumo-phonic coordination)	•Exhalation–phonation coordination	1.5′	5 repetitions
	•Production of long vowels	2.5′	5–10–15 * repetitions
	•Production of long vowels with pitch and volume variations	2.5′	5–10–15 * repetitions
Phonatory (for glottic occlusion)	•Hold the inhaled air as long as possible	1′	3 repetitions
	•Pushing exercise	5′	5–10–15 * repetitions
	•Harsh vowels attacks	5′	5–10–15 * repetitions
Resonance	•Velar stimulation (ice stimulation, veil massage)	1.5′	3 repetitions
	•Production of prolonged vowels	1′	5 repetitions
Articulatory	•Hyperarticulation	5′	3 repetitions
	•Articulatory exercises on single phonemes or syllables	5′	5–10–15 * repetitions
	•Verbal and non-verbal functional oral motor exercises	5′	5–10–15 * repetitions
	•Learning or articulatory positions	1′	3 repetitions
	•Speed control techniques	5′	5 repetitions
Prosodic	•Reading of affirmative, negative, interrogative, and exclamatory sentences	5′	3 repetitions
	•Vocalization with variable pitch and volume	5′	5–10–15 * repetitions
	•Reading of sentences with variable emphasis	5′	5–10–15 * repetitions

**Table 2:** Pre–post speech and language treatment.

Characteristics	T1	T2	*p*-Value
Barthel Index	71.4 ± 3.6	92.7 ± 5.4	<0.001 ^1^
RDP	153.4 ± 34.8	202.6 ± 23.4	<0.001 ^2^
IOPI Tongue	35.9 ± 7.6	45.2 ± 7.2	<0.001 ^2^
IOPI Lip Right	23.7 ± 7.1	28.0 ± 7.1	<0.001 ^2^
IOPI Lip Left	25.2 ± 7.4	29.1 ± 7.4	<0.001 ^2^
Vocal Intensity (dB)	53.5 ± 7.2	74.6 ± 6.6	<0.001 ^2^
IOPI Tongue Resistance	7.8 ± 2.3	13.1 ± 3.9	<0.001 ^2^

^1^ Wilcoxon test; ^2^ paired *t*-test; *p* value adjusted Bonferroni’s correction 0.007.

**Table 3:** Pre–post speech and language treatment (T1–T2) and follow up (T3).

Characteristics	T1	T2	T3	*p*-Value
VHI	42.4 ± 18.4	26.1 ± 17.0	34.0 ± 20.5	<0.001 ^1^
QoL–Dys	76.4 ± 35.8	47.7 ± 28.4	47.5 ± 27.7	<0.001 ^1^
PRAAT JITT	2.4 ± 1.4	1.4 ± 1.0	1.7 ± 0.9	0.021 ^2^
PRAAT SHIM	18.0 ± 0.6	14.4 ± 2.1	16.9 ± 1.2	<0.001 ^1^
MPT	10.8 ± 3.2	16.2 ± 5.0	13.1 ± 4.7	<0.001 ^2^

^1^ Friedman’s Anova with rank repeated measures; ^2^ Parametric Repeated Measures Anova (GLM); *p* value adjusted Bonferroni’s correction 0.005.

**Table 4:** Correlations between Iowa Oral Performance Instrument (IOPI) results.

Variables	IOPI Tongue (p)	IOPI Lip Right (p)	IOPI Lip Left (p)	IOPI Tongue Resistance (p)
Age	0.196 (0.300)	−0.169 (0.371)	0.102 (0.591)	−0.460 (0.010) ^+^
Years onset	−0.117 (0.537)	0.048 (0.799)	−0.005 (0.978)	−0.281 (0.133)
CIRS	−0.023 (0.902)	−0.347 (0.060)	0.177 (0.350)	−0.467 (0.009) ^+^
RDP	−0.076 (0.691)	−0.282 (0.131)	0.001 (0.996)	0.078 (0.683)
QoL-Dys score	0.038 (0.842)	0.325 (0.080)	0.025 (0.895)	0.206 (0.275)
PRAAT score	−0.223 (0.237)	−0.135 (0.477)	0.118 (0.536)	−0.071 (0.709)
VHI score	0.262 (0.162)	−0.248 (0.186)	−0.009 (0.996)	−0.101 (0.595)
Vocal Intensity (dB)	0.178 (0.346)	−0.085 (0.656)	0.134 (0.481)	−0.168 (0.376)
MoCA	0.116 (0.542)	0.508 (0.004) ^+^	−0.120 (0.528)	−0.032 (0.869)
Barthel Index	−0.091 (0.346)	−0.005 (0.978)	0.094 (0.622)	−0.059 (0.755)
MDS-UPDRS	0.053 (0.782)	−0.359 (0.051)	0.073 (0.700)	−0.465 (0.010) ^+^

^+^ for endurance measurements, the Spearman rho analysis was used.

## Data Availability

The datasets generated during the current study are available from the corresponding author upon reasonable request.
